# Supplementary material for: Structural and dynamic insights into the activation of the μ-opioid receptor by an allosteric modulator
Source: Nat Commun. 2024 May 13;15:3544. doi: 10.1038/s41467-024-47792-6 (PMC11091225; doi:10.1038/s41467-024-47792-6)
Supplement: Supplementary file 1 — Supplementary Information [file 41467_2024_47792_MOESM1_ESM.pdf]

Supplementary Information

Structural and Dynamic Insights into the Activation of the μ-Opioid Receptor by an Allosteric Modulator

Shun Kaneko[1,2], Shunsuke Imai[1], Tomomi Uchikubo-Kamo[1], Tamao Hisano[1], Nobuaki Asao[1,2], Mikako Shirouzu[1], and Ichio Shimada[1,3,*]

1. Center for Biosystems Dynamics Research (BDR), RIKEN, Kanagawa, Japan
2. Graduate School of Pharmaceutical Sciences, The University of Tokyo, Tokyo, Japan.
3. Graduate School of Integrated Science for Life, Hiroshima University, Hiroshima, Japan.

* Corresponding author

Ichio Shimada

RIKEN Center for Biosystems Dynamics Research, Tsurumi-ku, Yokohama 230-0045, Japan.

Phone: +81-45-503-7021

E-mail: ichio.shimada@riken.jp

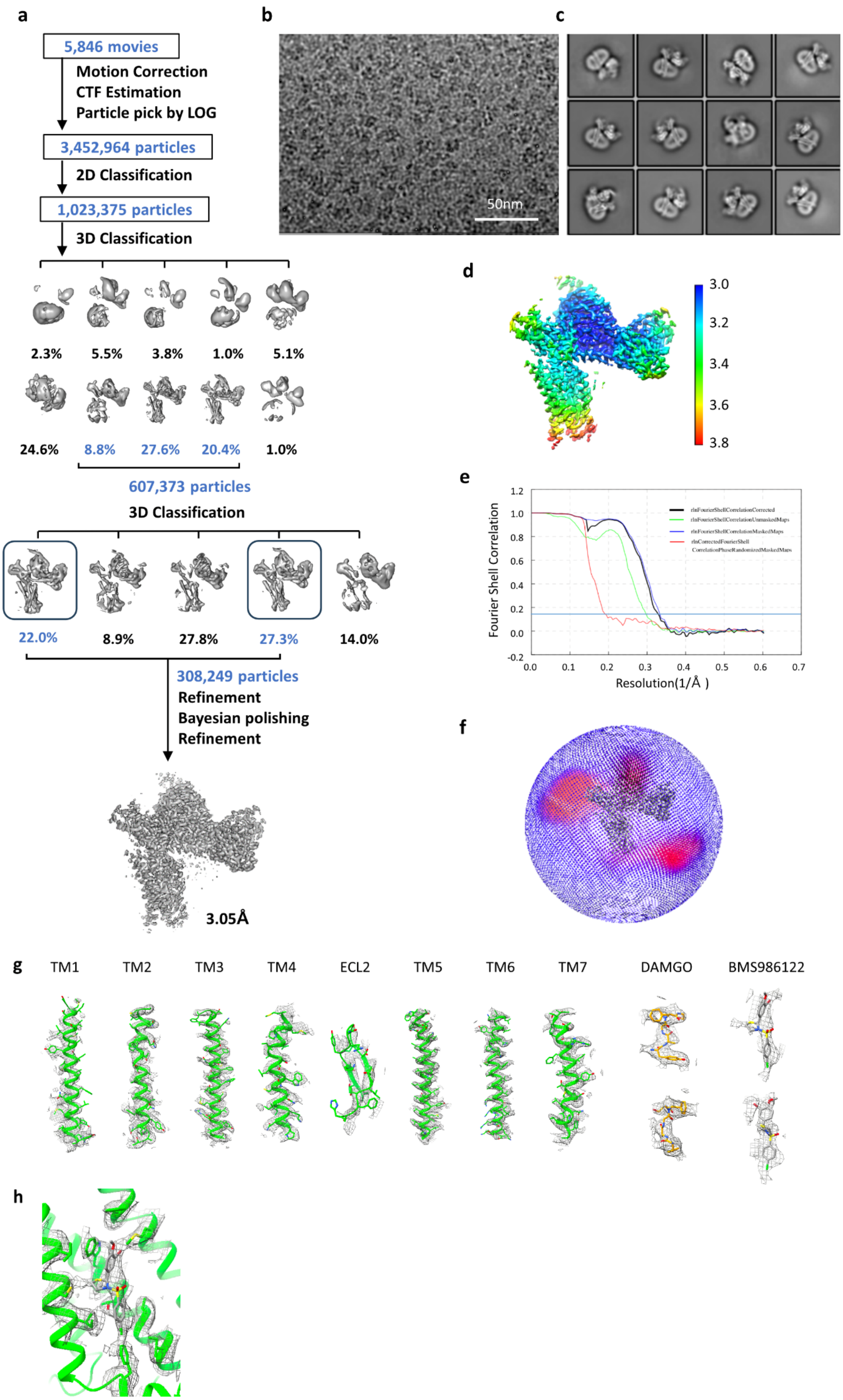

a
5,846 movies
Motion Correction
CTF Estimation
Particle pick by LOG
3,452,964 particles
2D Classification
1,023,375 particles
3D Classification
2.3%
5.5%
3.8%
1.0%
5.1%
24.6%
8.8%
27.6%
20.4%
1.0%
607,373 particles
3D Classification
22.0%
8.9%
27.8%
27.3%
14.0%
308,249 particles
Refinement
Bayesian polishing
Refinement
3.05Å
b
50nm
c
d
3.0
3.2
3.4
3.6
3.8
e
Fourier Shell Correlation
Resolution(1/Å)
f
g
TM1
TM2
TM3
TM4
ECL2
TM5
TM6
TM7
DAMGO
BMS986122
h

Supplementary Fig. 1 | Cryo-EM data acquisition and processing of the MOR(DAMGO)-G_i-scFv16 complex with BMS-986122.

a Flowchart for cryo-EM data processing of the complex structure.

b Representative raw micrograph.

c Gallery of two-dimensional class averages.

d Final three-dimensional density map colored by local resolution in side views.

e FSC curves after post-processing in RELION.

f Euler angle distribution of all particles included in the calculation of the final three-dimensional reconstruction.

g Cryo-EM density maps for all transmembrane helices, ECL2, DAMGO, and BMS-986122.

h Cryo-EM map of BMS-986122 and possible interacting residues.

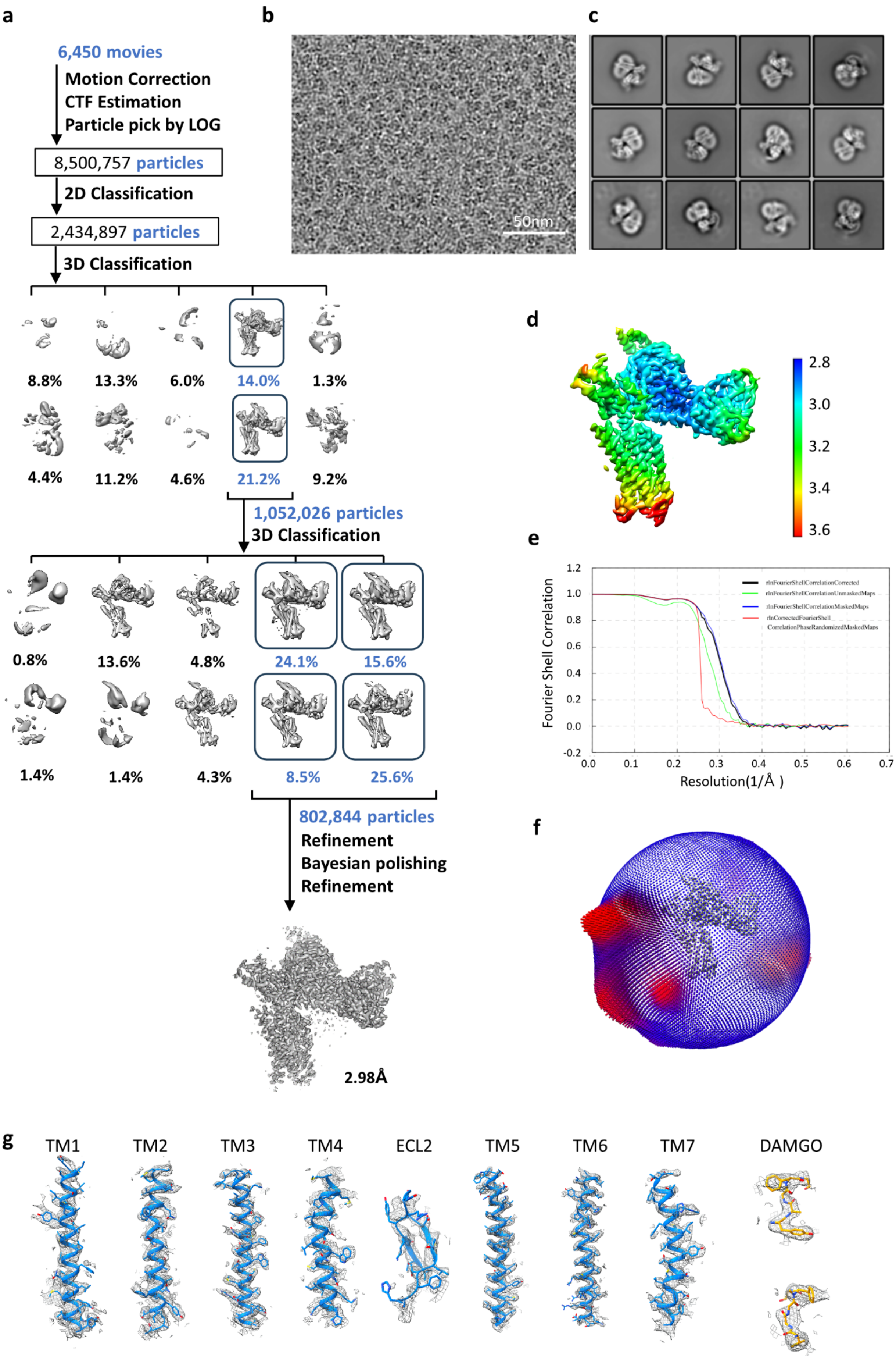
a
6,450 movies
Motion Correction
CTF Estimation
Particle pick by LOG
8,500,757 particles
2D Classification
2,434,897 particles
3D Classification
8.8%
13.3%
6.0%
14.0%
1.3%
4.4%
11.2%
4.6%
21.2%
9.2%
1,052,026 particles
3D Classification
0.8%
13.6%
4.8%
24.1%
15.6%
1.4%
1.4%
4.3%
8.5%
25.6%
802,844 particles
Refinement
Bayesian polishing
Refinement
2.98Å
b
50nm
c
d
2.8
3.0
3.2
3.4
3.6
e
Fourier Shell Correlation
Resolution(1/Å)
rlnFourierShellCorrelationCorrected
rlnFourierShellCorrelationUnmaskedMaps
rlnFourierShellCorrelationMaskedMaps
rlnCorrectedFourierShell CorrelationPhaseRandomizedMaskedMaps
f
g
TM1
TM2
TM3
TM4
ECL2
TM5
TM6
TM7
DAMGO

Supplementary Fig. 2 | Cryo-EM data acquisition and processing of the MOR(DAMGO)-G_i-scFv16 complex.

a Flowchart for cryo-EM data processing of the complex structure.

b Representative raw micrograph.

c Gallery of two-dimensional class averages.

d Final three-dimensional density map colored by local resolution in side views.

e FSC curves after post-processing in RELION.

f Euler angle distribution of all particles included in the calculation of the final three-dimensional reconstruction.

g Cryo-EM density maps for all transmembrane helices, ECL2, and DAMGO.

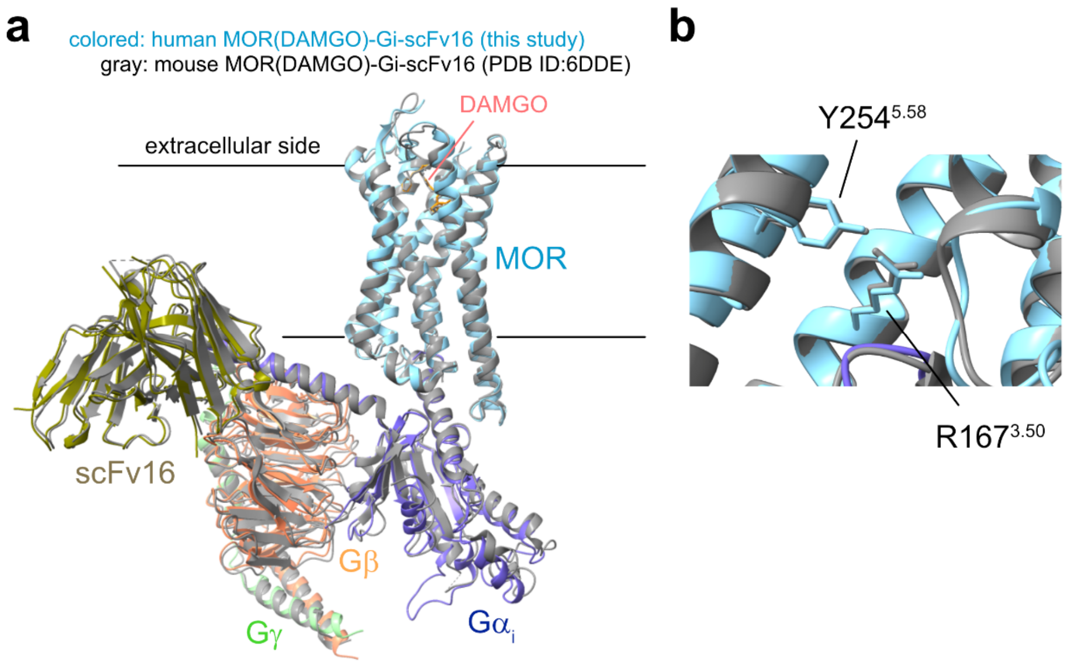

Supplementary Fig. 3 | Comparison of the structures of MOR(DAMGO)-G_i-scFv16

a Overlay of the two cryo-EM structures of MOR(DAMGO)-G_i-scFv16. The structure of the human MOR construct obtained in this study is colored, whereas the previous structure of the mouse MOR construct (PDB ID: 6DDE) is shown in gray. The r.m.s.d of Cα atoms of MOR molecules is 0.71 Å.

b A closeup view of Y254$^{5.58}$ and R167$^{3.50}$. Side chain atoms of the two structures are shown in stick models.

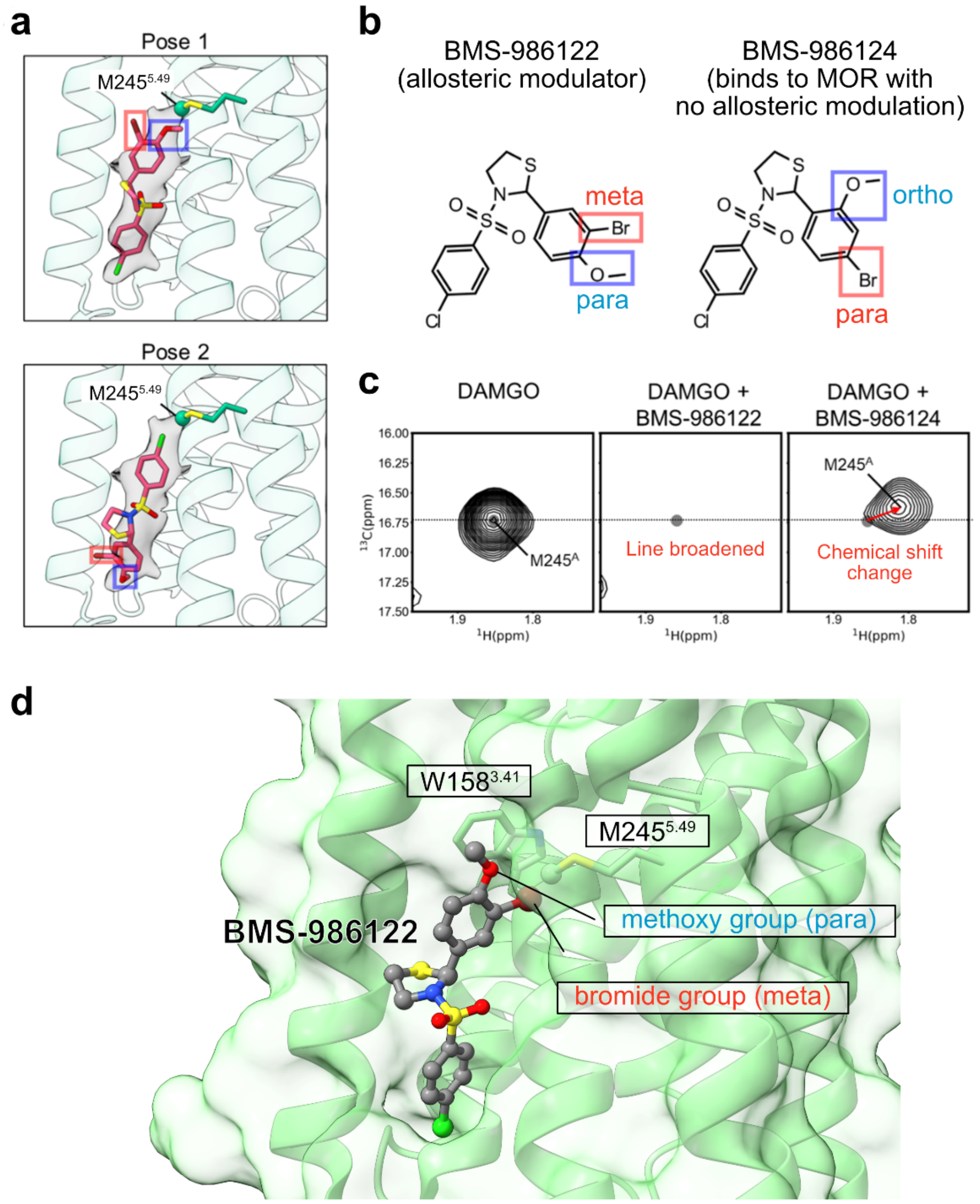

Supplementary Fig. 4 | Binding site and interaction of BMS-986122

a Two possible orientations of BMS-986122 to MOR. The Cε atom of M245$^{5.49}$ is represented by a sphere. The methoxy (blue) and bromo (red) groups are indicated by squares.

b The chemical structures of BMS-986122 (left) and BMS-986124 (right). The methoxy (blue) and bromo (red) groups are indicated by squares.

c ^{1}H-^{13}C HMQC signals of M245^{A} of MOR/Δ6M in DAMGO-bound (left), DAMGO and BMS-986122 bound (middle), and DAMGO and BMS-986124 bound (right) states. The M245^{A} signal was not observed for DAMGO and BMS-986122 bound MOR/Δ6M.

d A closeup view of BMS-986122 in the cryo-EM structure of MOR(DAMGO)-G$_i$-scFv16 docked using the information obtained from the NMR analyses (**b** and **c**). MOR structure model is shown as a transparent green surface with stick and cartoon models.

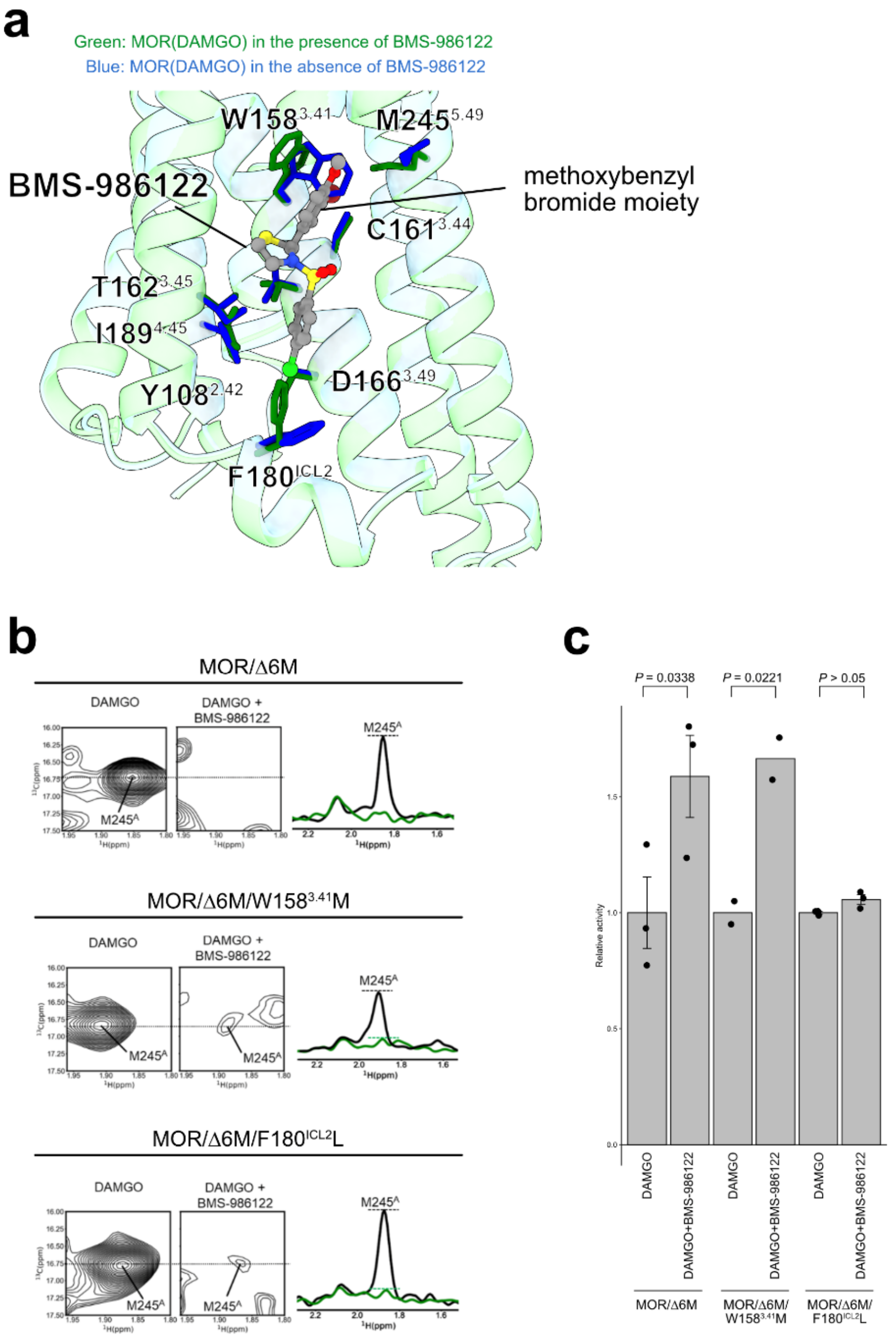

Supplementary Fig. 5 | Aromatic residues of MOR involved in interaction with BMS-986122

a Comparison of the binding site of BMS-986122 in the presence (green) and absence (blue) of BMS-986122.

b ^{1}H-^{13}C HMQC signals of M245^{A} and overlays of the cross section of the M245^{A} signal in the absence (black) and presence (green) of 100 μM BMS-986122. The M245^{A} signal was not observed for DAMGO and BMS-986122 bound MOR/Δ6M.

c Relative activities of MOR/Δ6M and its variants quantified by the GTP turnover assay. DAMGO:

Full agonist DAMGO-bound state in the absence of BMS-986122, DAMGO+BMS-986122: DAMGO-bound state in the presence of BMS-986122. Data are presented as mean ± standard error of the mean (s.e.m.) (n=3 independent replicates for MOR/Δ6M and MOR/Δ6M/F180^{ICL2}L, and n=2 independent replicates for MOR/Δ6M/W158$^{3.41}$M). Statistical significance was determined by a one-tailed Student's t-test, and *P* values are indicated. Source data are provided as a Source Data file.

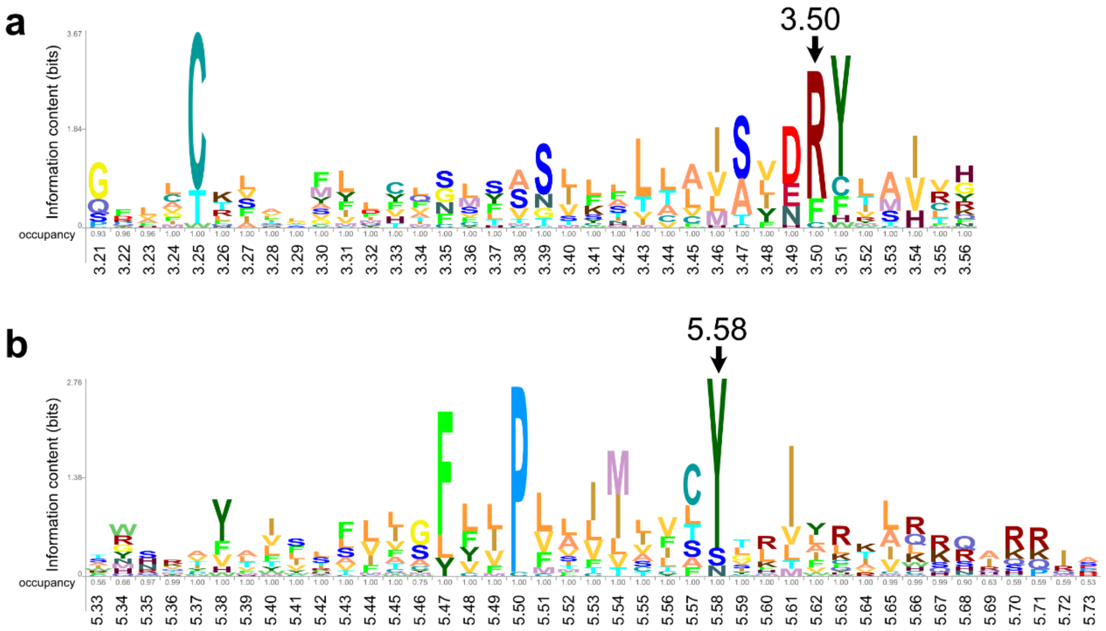

Supplementary Fig. 6 | Sequence conservation of class A GPCRs

Graphical representation of the sequence alignment of the 312 human class A GPCR proteins. Regions corresponding to TM3 (**a**) and TM5 (**b**) are shown. Positions 3.50 and 5.58 are highly conserved as arginine and tyrosine, respectively.

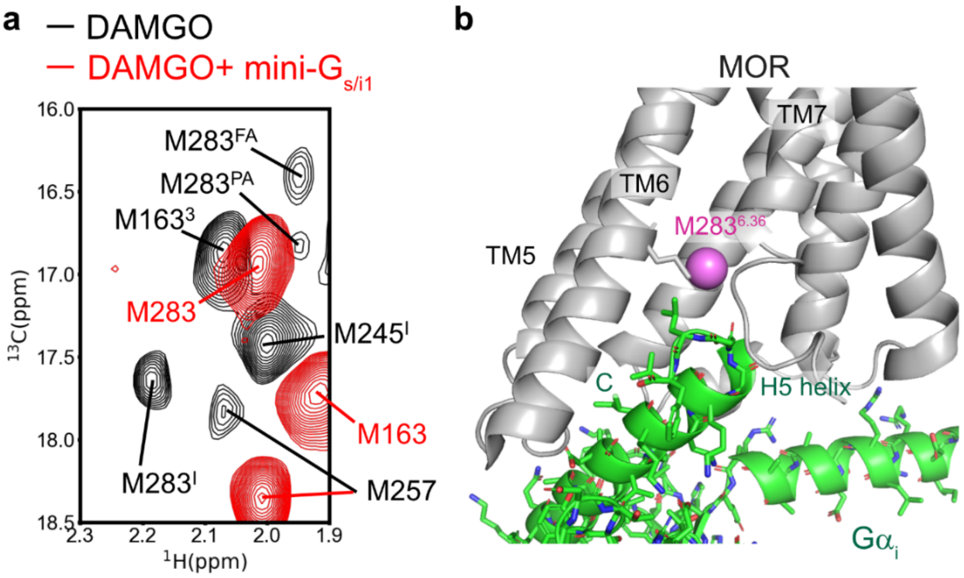

Supplementary Fig. 7 | ^{1}H-^{13}C HSQC spectra of MOR/Δ6M(DAMGO) in complex with mini-$G_{s/i1}$

a ^{1}H-^{13}C HMQC spectra of MOR/Δ6M(DAMGO). Black: MORΔ6M in the full agonist DAMGO-bound state, red: MORΔ6M in the full agonist DAMGO-bound state in complex with the engineered G_i protein, mini-$G_{s/i1}$. In the mini-$G_{s/i1}$ bound state, only one signal was observed from M283$^{6.36}$, indicating that the structural equilibrium among the fully-activated (FA), partially-activated (PA), and the inactivated (I) conformations is suppressed by the binding of mini-$G_{s/i1}$.

b A closeup view of the cryo-EM structure of MOR(DAMGO)-G_i-scFv16 (PDB ID: 6DDF). The Cε atom of M283$^{6.36}$ is shown as a violet sphere, which is on the interface with the $G\alpha_i$ in the trimeric G_i protein, indicating that the chemical shift of the methyl group would be directly affected by the binding of the G_i protein or mini-$G_{s/i1}$.

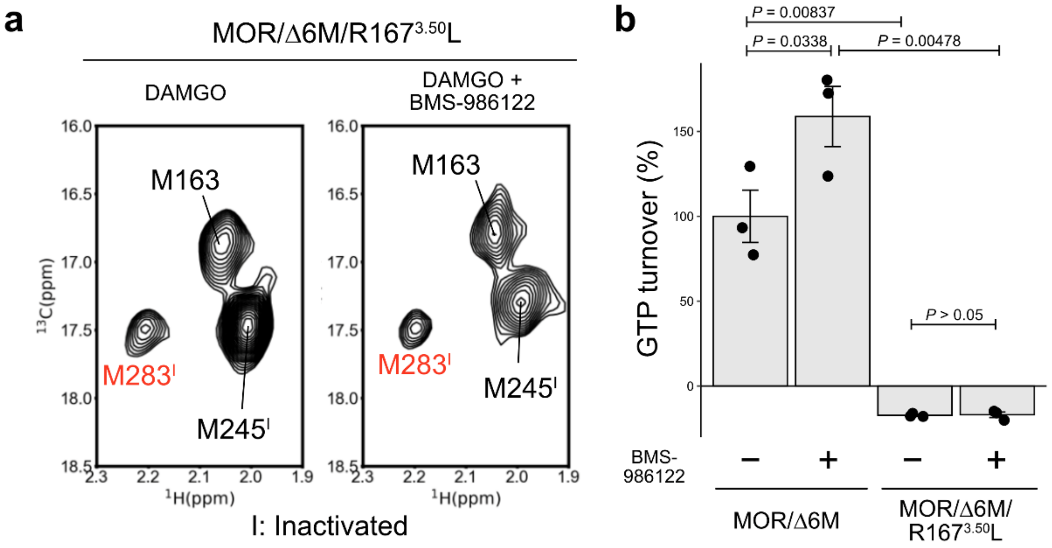

Supplementary Fig. 8 | Importance of the R167$^{3.50}$-Y254$^{5.58}$ interaction for structural dynamics and function of MOR investigated by the R167$^{3.50}$L variant.

a ^{1}H-^{13}C HMQC spectra of the R167$^{3.50}$L variant bound to DAMGO in the presence and absence of BMS-986122.

b GTP turnover of G_i protein stimulated by MOR/Δ6M or R167$^{3.50}$L variant in the DAMGO-bound state in the presence and absence of BMS-986122. All values were normalized by the GTP turnover rate of the G_i protein in the presence of MOR/Δ6M in the full agonist DAMGO-bound state (n=3 independent replicates). Data are presented as mean ± standard error of the mean (s.e.m.). Statistical significance was determined by a one-tailed Student's t-test, and *P* values are indicated. Source data are provided as a Source Data file.

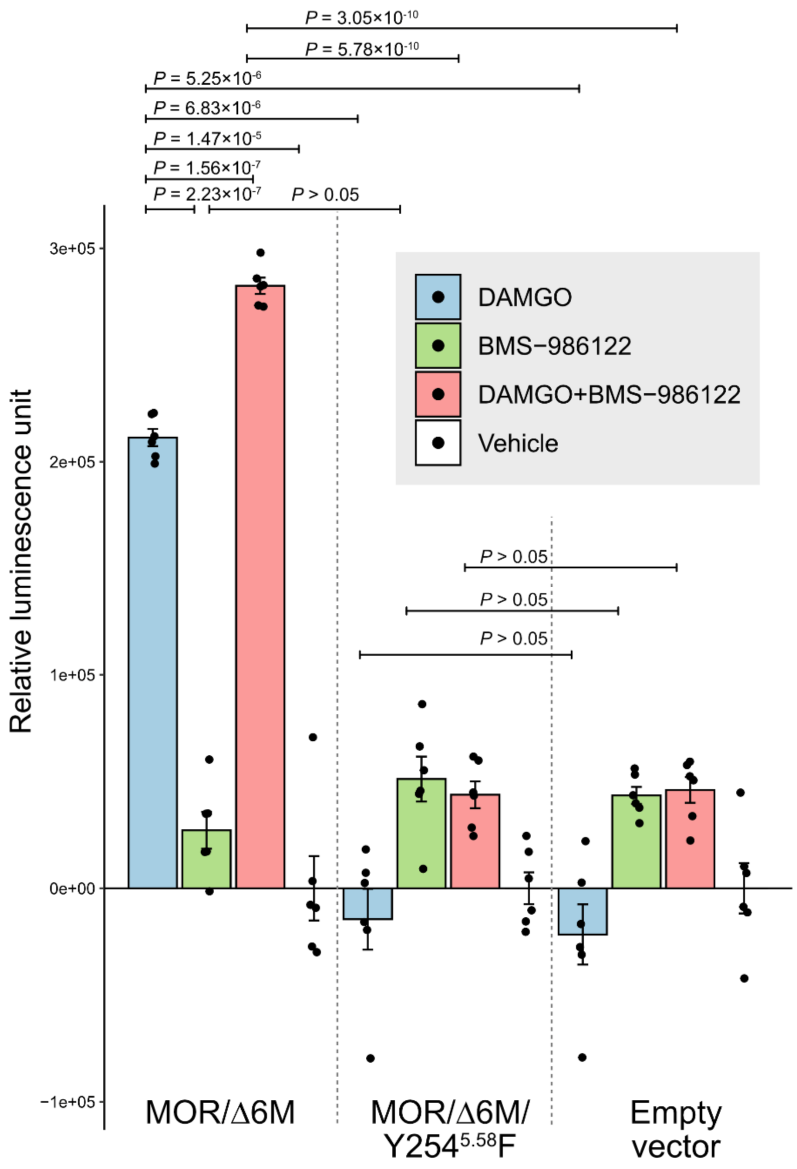

Supplementary Fig. 9 | cAMP inhibition assay for wild-type and Y254$^{5.58}$F variant of MOR/Δ6M
The degrees of G_i activation were quantified as the decrease in the magnitude of luminescence by the cAMP-dependent split luciferase, GloSensor (n=6 independent replicates). Data are presented as mean ± standard error of the mean (s.e.m.). Statistical significance was determined by a two-tailed unpaired Student's t-test, and *P* values are indicated. Source data are provided as a Source Data file.

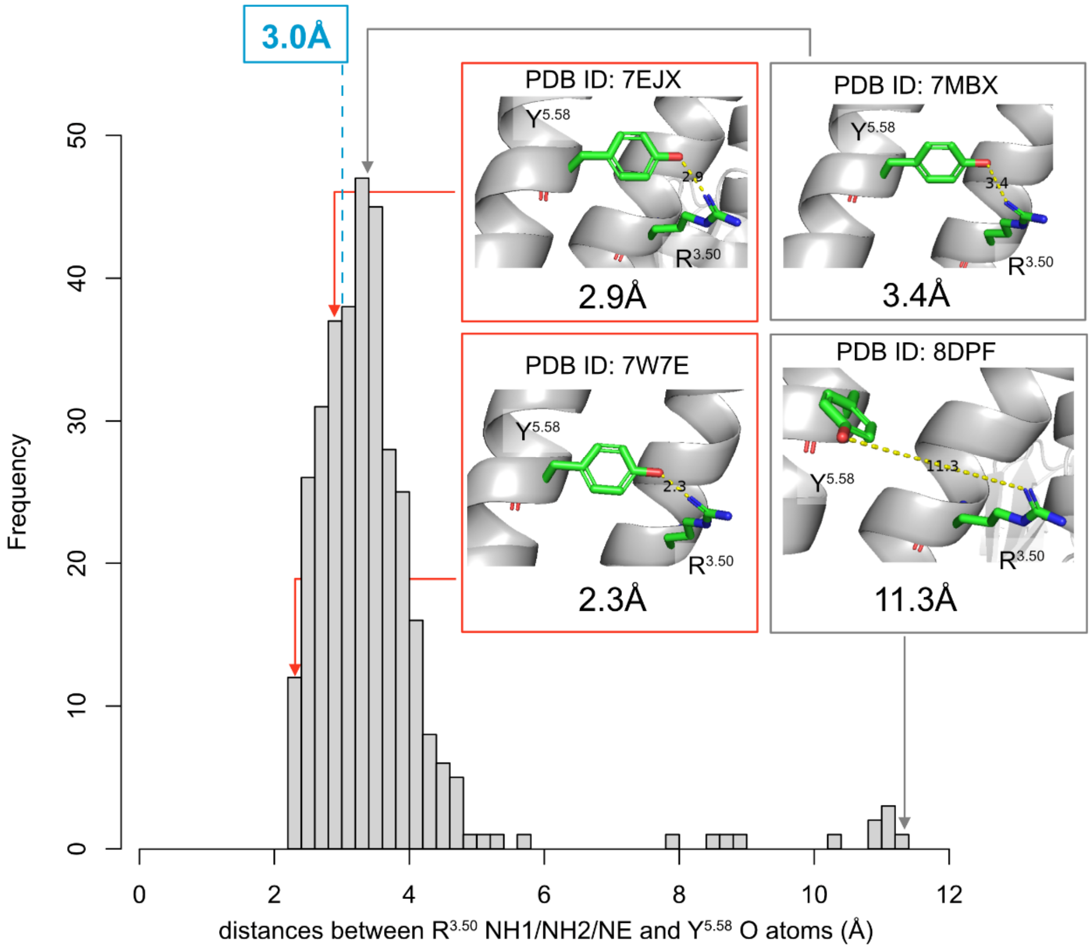

Supplementary Fig. 10 | Histogram of the distances between $R^{3.50}$ and $Y^{5.58}$ atoms in GPCR structures

A histogram of the distances between $R^{3.50}$ NH1/NH2/NE and $Y^{5.58}$ O atoms in class A GPCRs in complex with G proteins or mini-G proteins. For $R^{3.50}$, the closest nitrogen atom to the $Y^{5.58}$ O atom is selected for each structure. As of December 23rd, 2023, the GPCR database (https://gpcrdb.org/) lists 408 structures of GPCRs in complex with G proteins or mini-G proteins. Among them, 339 are of GPCRs harboring Arg at 3.50 and Tyr at 5.58. For these 339 structures, distances between the nitrogen atoms of $R^{3.50}$ in the side chain guanidino group, which can be the proton acceptor, and the oxygen atom of $Y^{5.58}$, which can be the proton donor, were calculated, and the smallest distance is used. Respective structures are shown as insets, Structures with possible hydrogen bonding between these two residues are highlighted in red boxes. Source data are provided as a Source Data file.

**Supplementary Table 1 | ** Cryo-EM data collection, refinement, and validation statistics

	**MOR(DAMGO)-G$_i$-scFv16 complex**	**MOR(DAMGO)-G$_i$-scFv16 containing BMS-986122**
EMDB ID	EMD-36989	EMD-36990
PDB ID	8K9K	8K9L
**Data collection and processing**		
Microsope	Titan Krios G4	Titan Krios G4
Magnification	105,000	105,000
Voltage (kV)	300	300
Detector	Gatan BioQuantum K3	Gatan BioQuantum K3
Energy filter	Gatan Quantum-LS, 15 eV slit	Gatan Quantum-LS, 15 eV slit
Movies	6450	5846
Electron dose (e-/Å^2)	50.5	50.5
Defocus range (μm)	−0.8 to −2.0	−0.8 to −2.0
Effective pixel size (Å)	0.83	0.83
Initial number of particles	8,500,757	3,452,964
Final number of particles	802,844	308,249
Symmetry imposed	C1	C1
Map resolution (Å)	2.98	3.05
FSC threshold	0.143	0.143
Map resolution range (Å)	2.8-3.8	3.0-3.8
**Refinement**		
Initial model used (PDB code)	7SBF, 6CRK	8K9K
Model resolution (Å)	2.98	3.05
FSC threshold	0.143	0.143
Map resolution range (Å)	2.8-3.8	3.0-3.8
Map sharpening *B*-factor (Å^2)	-128.43	-17.03
Nonhydrogen atoms	8,923	8,947
Protein residues	1,142	1,142
Mean *B*-factors (Å^2)		
Protein	57.04	82.85
Ligand	60.87	104.31
R.m.s. deviations		
Bond lengths (Å)	0.003	0.003
Bond angles (°)	0.624	0.584
**Validation**		
MolProbity score	1.63	1.58
Clash score	7.57	6.88
Rotamer outliers (%)	0.10	0.21
Ramachandran plot		
Favored (%)	96.62	96.80
Allowed (%)	3.38	3.20
Outliers (%)	0.00	0.00
