# Supplementary material for: Structural and dynamic insights into the activation of the μ-opioid receptor by an allosteric modulator
Source: Nat Commun. 2024 May 13;15:3544. doi: 10.1038/s41467-024-47792-6 (PMC11091225; doi:10.1038/s41467-024-47792-6)
Supplement: Supplementary file 3 — Reporting Summary [file 41467_2024_47792_MOESM3_ESM.pdf]

nature portfolio

Corresponding author(s): Ichio Shimada

Last updated by author(s): Apr 9, 2024

Reporting Summary

Nature Portfolio wishes to improve the reproducibility of the work that we publish. This form provides structure for consistency and transparency in reporting. For further information on Nature Portfolio policies, see our Editorial Policies and the Editorial Policy Checklist.

Statistics

For all statistical analyses, confirm that the following items are present in the figure legend, table legend, main text, or Methods section.

n/a	Confirmed	
[ ]	[x]	The exact sample size (*n*) for each experimental group/condition, given as a discrete number and unit of measurement
[ ]	[x]	A statement on whether measurements were taken from distinct samples or whether the same sample was measured repeatedly
[ ]	[x]	The statistical test(s) used AND whether they are one- or two-sided<br>*Only common tests should be described solely by name; describe more complex techniques in the Methods section.*
[x]	[ ]	A description of all covariates tested
[x]	[ ]	A description of any assumptions or corrections, such as tests of normality and adjustment for multiple comparisons
[ ]	[x]	A full description of the statistical parameters including central tendency (e.g. means) or other basic estimates (e.g. regression coefficient) AND variation (e.g. standard deviation) or associated estimates of uncertainty (e.g. confidence intervals)
[ ]	[x]	For null hypothesis testing, the test statistic (e.g. *F*, *t*, *r*) with confidence intervals, effect sizes, degrees of freedom and *P* value noted<br>*Give P values as exact values whenever suitable.*
[x]	[ ]	For Bayesian analysis, information on the choice of priors and Markov chain Monte Carlo settings
[x]	[ ]	For hierarchical and complex designs, identification of the appropriate level for tests and full reporting of outcomes
[x]	[ ]	Estimates of effect sizes (e.g. Cohen's *d*, Pearson's *r*), indicating how they were calculated

Our web collection on statistics for biologists contains articles on many of the points above.

Software and code

Policy information about availability of computer code

Data collection	Topspin 3.5, Topspin 4.0
Data analysis	RELION-3.1.3, MotionCor2, CTFFIND 4.1, crYOLO 1.9.7, UCSF ChimeraX 1.7, Coot 1.1, phenix.real_space.refine 1.21, eLBOW in Phenix 1.21, Phenix 1.21, AutoDock Vina 1.2.5

For manuscripts utilizing custom algorithms or software that are central to the research but not yet described in published literature, software must be made available to editors and reviewers. We strongly encourage code deposition in a community repository (e.g. GitHub). See the Nature Portfolio guidelines for submitting code & software for further information.

Data

Policy information about availability of data

All manuscripts must include a data availability statement. This statement should provide the following information, where applicable:

- Accession codes, unique identifiers, or web links for publicly available datasets
- A description of any restrictions on data availability
- For clinical datasets or third party data, please ensure that the statement adheres to our policy

The cryo-EM density map data generated in this study have been deposited in the Electron Microscopy Data Bank (EMDB) database under accession codes EMD-36990 [https://www.ebi.ac.uk/pdbe/entry/emdb/EMD-36990] (MOR(DAMGO)-Gi-scFv16 complex in the presence of BMS-986122) and EMD-36989 [https://www.ebi.ac.uk/pdbe/entry/emdb/EMD-36989] (that in the absence of BMS-986122). The atomic coordinate data generated in this study have been deposited in the PDB database under accession code 8K9L [http://doi.org/10.2210/pdb8K9L/pdb] (MOR(DAMGO)-Gi-scFv16 complex in the presence of BMS-986122) and 8K9K

[http://doi.org/10.2210/pdb8K9K/pdb] (that in the absence of BMS-986122). Other structure data used in this study are available in PDB under accession codes 7SBF [http://doi.org/10.2210/pdb7SBF/pdb], 4DKL [http://doi.org/10.2210/pdb4DKL/pdb], 6CRK [http://doi.org/10.2210/pdb6CRK/pdb], 6DDE [http://doi.org/10.2210/pdb6DDE/pdb], and 6DDF [http://doi.org/10.2210/pdb6DDF/pdb]. Source data are provided as a Source Data file.

Research involving human participants, their data, or biological material

Policy information about studies with human participants or human data. See also policy information about sex, gender (identity/presentation), and sexual orientation and race, ethnicity and racism.

Reporting on sex and gender	N/A
Reporting on race, ethnicity, or other socially relevant groupings	N/A
Population characteristics	N/A
Recruitment	N/A
Ethics oversight	N/A

Note that full information on the approval of the study protocol must also be provided in the manuscript.

Field-specific reporting

Please select the one below that is the best fit for your research. If you are not sure, read the appropriate sections before making your selection.

☒ Life sciences ☐ Behavioural & social sciences ☐ Ecological, evolutionary & environmental sciences

For a reference copy of the document with all sections, see nature.com/documents/nr-reporting-summary-flat.pdf

Life sciences study design

All studies must disclose on these points even when the disclosure is negative.

Sample size	No statistical methods were used to predetermine sample size. Sample sizes were determined based on experimental trials to allow for confident statistical tests. Cryo-EM data sample size was chosen based on the sufficient number of particles to obtain the high resolution constructions.
Data exclusions	No data were excluded from the analyses.
Replication	Three or more replicates were used for each sample, except for the GTP turnover assay for W158M, where the experiments were replicated twice. All replicates were successful. The repeat numbers are indicated in the figure legends.
Randomization	For cryo-EM analysis, particles were randomly assigned to half-maps for resolution estimation. Randomization method was not utilized to the other experiments because the samples in this study were not allocated into experimental groups during data acquisition and analysis.
Blinding	Blinding is not relevant for this study because no group allocation was performed.

Reporting for specific materials, systems and methods

We require information from authors about some types of materials, experimental systems and methods used in many studies. Here, indicate whether each material, system or method listed is relevant to your study. If you are not sure if a list item applies to your research, read the appropriate section before selecting a response.

Materials & experimental systems

n/a	Involved in the study	
☒	☐	Antibodies
☐	☒	Eukaryotic cell lines
☒	☐	Palaeontology and archaeology
☒	☐	Animals and other organisms
☒	☐	Clinical data
☒	☐	Dual use research of concern
☒	☐	Plants

Methods

n/a	Involved in the study	
☒	☐	ChIP-seq
☒	☐	Flow cytometry
☒	☐	MRI-based neuroimaging

Eukaryotic cell lines

Policy information about cell lines and Sex and Gender in Research

Cell line source(s)	HEK293T cell line was purchased from KAC Co.,Ltd.
Authentication	The cell lines were authenticated by the supplier by using mycoplasma analysis, sterility analysis, and visual analysis forgrowth determination, morphology and viability.
Mycoplasma contamination	All cell lines tested negative for mycoplasma contamination.
Commonly misidentified lines (See ICLAC register)	No commonly misidentified cell lines were used.

Plants

Seed stocks	N/A
Novel plant genotypes	N/A
Authentication	N/A
